# Aqueous humour interleukin-6 and vision outcomes with anti-vascular endothelial growth factor therapy

**DOI:** 10.1038/s41433-024-03015-2

**Published:** 2024-04-15

**Authors:** Yasir Jamal Sepah, Diana V. Do, Marina Mesquida, Bann-Mo Day, Steven Blotner, Rubbia Afridi, Muhammad Sohail Halim, Kyu Hong, Eric Wakshull, Sascha Fauser, Ivaylo Stoilov, Quan Dong Nguyen, on behalf of the HARBOR, P. Abraham, D. V. Alfaro, A. Antoszyk, M. Antworth, B. Baker, C. Baker, M. Balles, D. Boyer, W. Bridges, D. M. Brown, B. Busbee, M. Busquets, C. Chan, N. Chaudhry, S. Chen, J. Christoforidis, T. Ciulla, W. L. Clark, T. Cleland, T. Connor, A. Daccache, A. Dessouki, K. Diddie, B. Doft, R. Dreyer, D. W. Faber, L. Feiner, R. Feldman, P. Ferrone, G. Fox, S. Foxman, R. Frenkel, A. Fung, R. Gallemore, T. Ghuman, V. Gonzalez, A. Gordon, C. Gordon, S. Gupta, S. Hariprasad, J. Heier, A. Ho, D. Holmes, J. Huang, J. P. Hubschman, H. Hudson, D. Ie, R. Johnson, R. Katz, S. Kiss, J. Kitchens, G. Kokame, E. Lit, M. Liu, J. K. Luu, M. MacCumber, S. Madreperla, D. Marcus, A. Martidis, J. Martinez, M. Michels, D. Miller, L. Morse, M. Nasir, Q. Nguyen, S. Oliver, K. Olsen, S. Patel, P. Pavan, J. Pearlman, J. Prenner, C. Regillo, E. Reichel, R. Rosa, S. Rose, S. Sadda, M. Samuel, L. Singerman, M. Singer, R. Singh, G. Stoller, I. Suner, A. Tabassian, B. Taney, A. Thach, M. Thomas, M. Tolentino, D. Tom, P. Tornambe, R. Torti, S. Truong, T. Verstraeten, A. Wagner, K. Wald, P. Weber, P. Weishaar, M. Wieland, D. Williams, T. Wong, M. Wood, J. Wroblewski, K. Zhang, READ-3 Investigators, D. V. Do, E. Lit, E. Kruger, J. Pollack, L. Halperin, M. Bennett, D. Boyer, D. Callanan, K. Zhang, A. Symons, P. Abraham

**Affiliations:** 1 Byers Eye Institute, Spencer Center for Vision Research, Stanford University, Palo Alto, CA USA; 2 Roche Pharma Research and Early Development, Basel, Switzerland; 3 Genentech Inc., South San Francisco, CA USA; 4 Ocular Imaging Research and Reading Center, Sunnyvale, CA USA; 5 Black Hills Regional Eye Institute, Rapid City, SD USA; 6 Charleston Neuroscience Institute, Ladson, SC USA; 7 Southeast Clinical Research, Charlotte, NC USA; 8 Retina Consultants Of Carolina, Greenville, SC USA; 9 Vitreo-Retinal Associates Of Worcester, Worcester, MA USA; 10 Paducah Retinal Center, Paducah, KY USA; 11 Retina Center of Maine, Portland, ME USA; 12 Retina -Vitreous Associates Medical Group, Beverly Hills, CA USA; 13 Western Carolina Retinal Associates PA, Asheville, NC USA; 14 Retina Consultants of Houston, Houston, TX USA; 15 Tennessee Retina PC, Nashville, TN USA; 16 Associates in Ophthalmology, West Mifflin, PA USA; 17 Southern CA Desert Retina Consultants, Palm Desert, CA USA; 18 New England Retina Associates, New London, CT USA; 19 Orange County Retina Medical Group, Santa Ana, CA USA; 20 OSU Eye Physicians & Surgeons, Columbus, OH USA; 21 Midwest Eye Institute, Indianapolis, IN USA; 22 Palmetto Retina Center, West Columbia, SC USA; 23 Retina Associates of South Texas, San Antonio, TX USA; 24 Medical College of Wisconsin, Milwaukee, WI USA; 25 Danbury Eye Physicians & Surgeons, Danbury, CT USA; 26 Retinal Diagnostic Center, Campbell, CA USA; 27 Retinal Consultants of Southern California, Westlake Village, CA USA; 28 Retina-Vitreous Consultants, Pittsburgh, PA USA; 29 Retina Northwest, Portland, OR USA; 30 Rocky Mountain Retina Consultants, Salt Lake City, UT USA; 31 Retina Associates of New Jersey, Teaneck, NJ USA; 32 Florida Eye Clinic, Altamonte Springs, FL USA; 33 Vitreoretinal Consultants, Great Neck, NY USA; 34 Retina Associates, Shawnee Mission, KS USA; 35 Retinal and Ophthalmic Consultants PC, Northfield, NJ USA; 36 East Florida Eye Institute, Stuart, FL USA; 37 Pacific Eye Associates, San Francisco, CA USA; 38 Retina Macula Institute, Torrance, CA USA; 39 Retina Consultants of Southwest Florida, Fort Myers, FL USA; 40 Valley Retina Institute PA, McAllen, TX USA; 41 Associated Retina Consultants, Phoenix, AZ USA; 42 TLC Laser and Eye Care Centers, Jackson, MI USA; 43 Retina Specialty Institute, Pensacola, FL USA; 44 University of Chicago, Chicago, IL USA; 45 Ophthalmic Consultants of Boston, Boston, MA USA; 46 Wills Eye Institute, Philadelphia, PA USA; 47 Wyoming Retina Associates PC, Casper, WY USA; 48 Yale University Eye Center, New Haven, CT USA; 49 Jules Stein Eye Institute, Los Angeles, CA USA; 50 Retina Centers PC, Tucson, AZ USA; 51 Delaware Valley Retina Associates, Lawrenceville, NJ USA; 52 West Coast Retina Medical Group Inc, San Francisco, CA USA; 53 Florida Eye Microsurgical Institute, Boynton Beach, FL USA; 54 Weill Cornell Medical College, New York, NY USA; 55 Retina Associates, Lexington, KY USA; 56 The Retina Center at Pali Momi, Aiea, HI USA; 57 East Bay Retina Consultants, Oakland, CA USA; 58 Colorado Retina Associates PC, Golden, CO USA; 59 Retina Consultants of Southern Colorado PC, Colorado Springs, CO USA; 60 Illinois Retina Associates SC, Oak Park, IL USA; 61 Retina Associates of New Jersey, Vauxhall, NJ USA; 62 Southeast Retina Center, Augusta, GA USA; 63 Miramar Eye Specialists, Ventura, CA USA; 64 Austin Retina Associates, Austin, TX USA; 65 Retina Care Specialists, Palm Beach Gardens, FL USA; 66 Cincinnati Eye Institute, Cincinnati, OH USA; 67 University of California Davis Medical Center, Sacramento, CA USA; 68 California Retina Consultants, Santa Barbara, CA USA; 69 Wilmer Eye Institute, Baltimore, MD USA; 70 Rocky Mountain Lions Eye Institute, Aurora, CO USA; 71 Retina Vitreous Consultants, Johnstown, PA USA; 72 West Texas Retina Consultants PA, Abilene, TX USA; 73 University of South Florida Eye Institute, Tampa, FL USA; 74 Retina Center of Chico Inc., Sacramento, CA USA; 75 Retina Vitreous Center, Princeton, NJ USA; 76 Mid-Atlantic Retina, Huntingdon Valley, PA USA; 77 Tufts - New England Medical Center, Boston, MA USA; 78 Scott and White Hospital, Temple, TX USA; 79 Retina Associates of Western New York, Rochester, NY USA; 80 Doheny Eye Institute, Los Angeles, CA USA; 81 Retina Institute of California, Arcadia, CA USA; 82 Retina Associates of Cleveland Inc, Beachwood, OH USA; 83 Medical Center Ophthalmology Associates, San Antonio, TX USA; 84 Cleveland Clinic Foundation, Cleveland, OH USA; 85 Ophthalmic Consultants of Long Island, Lynbrook, NY USA; 86 Retina Associates of Florida PA, Tampa, FL USA; 87 Retina Institute of Virginia, Richmond, VA USA; 88 Retina Group of Florida, Fort Lauderdale, FL USA; 89 Retina Consultants of Nevada, Las Vegas, NV USA; 90 Barnes Retina Institute, Saint Louis, MO USA; 91 Center for Retina and Macular Disease, Winter Haven, FL USA; 92 New England Retina Associates, Hamden, CT USA; 93 Retina Consultants San Diego, Poway, CA USA; 94 Retina Specialists, Desoto, TX USA; 95 Pennsylvania Retinal Specialists, Camp Hill, PA USA; 96 Allegheny General Hospital, Pittsburgh, PA USA; 97 Wagner Mandell, Virginia Beach, VA USA; 98 Retina Associates of New York, New York, NY USA; 99 Island Retina, Shirley, NY USA; 100 Vitreo-Retinal Consultants, Wichita, KS USA; 101 Northern California Retina Vitreous Associates, Mountain View, CA USA; 102 Vitreo Retinal Surgery PA, Edina, MN USA; 103 Retina Consultants of Houston, The Woodlands, TX USA; 104 Eye Surgical Associates, Lincoln, NE USA; 105 Cumberland Valley Retina PC, Hagerstown, MD USA; 106 UC San Diego Shiley Eye Center, La Jolla, CA USA; 107 Wilmer Eye Institute, Johns Hopkins University, Baltimore, MD USA; 108 East Bay Retina Institute, Oakland, CA USA; 109 Eye Care Specialists, Kingston, PA Canada; 110 Illinois Retina Associates, Chicago, IL USA; 111 Retina Institute of Hawaii, Honolulu, HI USA; 112 Retina Vitreous Associates, Beverly Hills, CA USA; 113 Texas Retina Associates, Arlington, TX USA; 114 Shiley Eye Center, University of California, San Diego, San Diego, CA USA; 115 University of Kansas, Kansas City, KS USA; 116 Black Hills Eye Institute, Rapid City, SD USA

**Keywords:** Medical research, Diseases

## Abstract

**Background:**

This analysis evaluated aqueous humour (AH) interleukin (IL)-6 concentrations and the association between AH IL-6 and visual outcomes in patients with neovascular age-related macular degeneration (nAMD) or diabetic macular oedema (DMO) receiving anti–vascular endothelial growth factor (VEGF) monotherapy.

**Methods:**

Post hoc analysis of the multicentre, double-masked, randomised HARBOR (NCT00891735) and READ-3 (NCT01077401) trials. HARBOR enrolled treatment-naïve nAMD patients. READ-3 enrolled treatment-naïve/previously treated DMO patients. HARBOR patients received ranibizumab 0.5 or 2.0 mg monthly or as needed; AH samples were collected at month 2, after two previous intravitreal injections. READ-3 patients received ranibizumab 0.5 or 2.0 mg as needed; AH samples were collected at baseline and months 3, 6, 9, and 12. Main outcome measure: association between AH IL-6 concentrations and month 24 best-corrected visual acuity (BCVA).

**Results:**

In both trials (HARBOR, *N* = 36; READ-3, *N* = 137), patients with higher AH IL-6 concentrations had worse visual outcomes. HARBOR patients with low AH IL-6 concentrations at month 2 had a mean (95% CI) BCVA change at month 24 of +2.9 (−2.6, 8.3) letters, whereas patients with high AH concentrations had a mean (95% CI) BCVA change of −9.0 (−22.7, 4.7) letters. READ-3 patients with low AH concentrations at baseline had a mean (95% CI) BCVA change at month 12 of +9.3 (7.4, 11.3) letters, whereas patients with high AH concentrations had a mean (95% CI) BCVA change of +5.6 (2.2, 9.1) letters.

**Conclusions:**

Higher IL-6 AH concentrations may predict suboptimal visual responses to anti–VEGF monotherapy in patients with nAMD/DMO.

## Introduction

Interleukin-6 (IL-6) is a pro-inflammatory cytokine with pleiotropic activity that plays a central role in host defense against environmental stress, such as infection and tissue injury [1]. Dysregulated IL-6 production has been implicated in the pathogenesis of various ocular conditions. Indeed, significantly elevated IL-6 concentrations have been detected in aqueous humour (AH) or vitreous samples from patients with various retina diseases, including diabetic retinopathy, diabetic macular oedema (DMO), neovascular age-related macular degeneration (nAMD), retinal vein occlusion, uveitis, and uveitic macular oedema [2–6]. Interestingly, intraocular concentrations of vascular endothelial growth factor (VEGF) and IL-6 have been reported to be correlated with diabetic retinopathy and DMO severity [7–9]. Further, in nAMD, AH IL-6 concentrations have been associated with several measures of disease activity, including the height of retinal pigment epithelium detachment [10], macular thickness and volume [11], and choroidal neovascularisation size [12]. In addition, there is a growing body of evidence on the off-label use of the systemic IL-6 receptor inhibitor tocilizumab for noninfectious uveitis and uveitic macular oedema [13], including STOP-UVEITIS, which showed significant improvement and/or complete resolution of macular oedema after multiple intravenous infusions [14, 15].

From the preclinical perspective, in murine models, IL-6 inhibition through antibody-based blockade of IL-6 or its receptor were found to suppress laser-induced choroidal neovascularisation [16, 17]. Moreover, IL-6 blockade inhibits signal transducer and activator of transcription-3 pathway activation and the expression of other important mediators, including VEGF [16, 18]. Similarly, IL-6 has been implicated in the pathogenesis of ocular disease in various in vitro models, in which IL-6 was shown to induce VEGF expression and enhance endothelial permeability [19–22]. Collectively, these findings implicate IL-6 signaling in a variety of retinal diseases, including nAMD and DMO, and identify IL-6 as a potential specific therapeutic target in these conditions.

Previous studies have demonstrated correlations between AH IL-6 concentrations and changes in central retinal thickness in response to anti-VEGF therapy, suggesting that intraocular IL-6 levels may predict anatomical responses to treatment [3, 23]. However, the relationship between intraocular IL-6 and functional outcomes, including visual acuity (VA), achieved with anti-VEGF therapy for nAMD and DMO is currently unclear. This post hoc analysis of the HARBOR and READ-3 trials aimed to quantify AH IL-6 concentrations in patients with nAMD and DMO, respectively, and to examine the association between intraocular IL-6 and visual responses to ranibizumab treatment.

## Methods

### HARBOR and READ-3

This was a post hoc analysis of the phase 3 HARBOR trial (ClinicalTrials.gov identifier: NCT00891735) and the phase 2 READ-3 trial (NCT01077401)—two multicentre, double-masked, randomized studies investigating ranibizumab in patients with nAMD and DMO, respectively. Full details of the study design, patient population, treatment protocol, and pre-specified outcome measures for HARBOR and READ-3 are described in their respective trial publications [24, 25]. In HARBOR, 1097 treatment-naïve patients with subfoveal nAMD and best-corrected visual acuity (BCVA) 20/40–20/320 Snellen equivalent were randomized to intravitreal ranibizumab 0.5 mg or 2.0 mg, administered monthly or as needed (pro re nata, after 3 monthly initiation doses) through month 24 [24]. In READ-3, 152 treatment-naïve or previously treated patients with center-involved DMO and BCVA 20/40–20/320 Snellen equivalent were randomized to intravitreal ranibizumab 0.5 mg or 2.0 mg, administered pro re nata (after 6 monthly initiation doses) through month 24 [25]. No patients in either trial had active autoimmune disease. One patient in READ-3 underwent a cataract extraction during the first 12 months of the trial. Both trials adhered to the tenets of the Declaration of Helsinki and the Health Insurance Portability and Accountability Act. Protocols were approved by institutional review boards, ethics committees, or as applicable, and all patients provided written informed consent to participate.

### VA and IL-6 measurement

Pre-specified efficacy measures in HARBOR and READ-3 included VA outcomes over the trial period [24, 25]. Therefore, BCVA (measured in Early Treatment Diabetic Retinopathy Study [ETDRS] letters) was prospectively assessed in all patients at monthly study visits from baseline through month 24.

In HARBOR, optional AH samples were collected at the month 2 study visit (immediately preceding the third intravitreal injection). This analysis included patients with available matching serum samples. IL-6 concentrations were quantified using an IMPACT Assay System (Roche Diagnostics GmbH, Penzberg, Germany), with a sensitivity of 1 pg/mL. In READ-3, optional serial AH samples were collected prospectively at baseline and months 3, 6, 9, and 12; IL-6 concentrations were analysed using an MSD MULTI-SPOT Cytokine Assay System (Meso Scale Diagnostics, LLC, Rockville, MD, USA), with a sensitivity of 4 pg/mL.

### Post hoc analyses

Post hoc analyses were carried out to determine serum and/or AH concentrations of IL-6 in patients with nAMD (HARBOR) and DMO (READ-3) and to investigate the possible association between IL-6 and vision outcomes achieved with ranibizumab in these patients (note: serum IL-6 concentration data were not available for READ-3). For HARBOR, a two-sided Wilcoxon rank sum test was used to detect differences in IL-6 concentrations between serum and AH samples at month 2. The relationship between these variables was assessed using the Pearson correlation coefficient. In addition, we examined the mean change in BCVA from month 2 through month 24 in patients with month 2 AH IL-6 concentrations ≥15 pg/mL (representing the top 25% of patients; categorised as high concentrations) and <15 pg/mL (representing the bottom 75% of patients; categorised as low concentrations). For READ-3 patients, IL-6 concentrations in serial AH samples were measured from baseline through month 12 to assess the effect of anti-VEGF therapy over time. Spearman correlation coefficients were calculated to evaluate the correlation between AH IL-6 concentrations and BCVA at each time point. The mean change in BCVA from baseline through month 12 in READ-3 was examined in patients with baseline AH IL-6 concentrations ≥46.49 pg/mL (representing the top 25% of patients; categorised as high concentrations) and <46.49 pg/mL (representing the bottom 75% of patients; categorised as low concentrations).

## Results

### Patient disposition

The present analysis included 36 (ranibizumab 0.5 mg: *n* = 16; 2.0 mg: *n* = 20) patients with nAMD from HARBOR and 137 (0.5 mg: *n* = 69; 2.0 mg: *n* = 68) patients with DMO from READ-3. Baseline characteristics for these subgroups were similar to those for the respective trial populations (Table 1).

**Table 1:** Key baseline characteristics of HARBOR and READ-3 patients included in the IL-6 analyses.

	HARBOR	
	Overall population (*N* = 1097)	IL-6 subgroup (*n* = 36)
Age, mean (SD), *y*	78.7 (8.3)	79.9 (6.3)
White, *n* (%)	1061 (97)	36 (100)
Female, *n* (%)	651 (59)	22 (61)
BCVA (ETDRS letters), mean (SD)	53.9 (12.8)	56.3 (12.3)
CFT, mean (SD), µm	344.3 (142.9)	346.3 (119.6)
	**READ-3**	
	2.0 mg ranibizumab (*n* = 68)	0.5 mg ranibizumab (*n* = 69)
Age, mean (SD), *y*	64.0 (9.3)	65.3 (9.9)
White, *n* (%)	35 (63.6)	40 (74.1)
Female, *n* (%)	33 (48.5)	28 (40.6)
BCVA (ETDRS letters), mean (SD)	29.0 (11.3)	27.1 (11.0)
CST, mean (SD), µm	431.2 (116.3)	444.0 (138.6)

*BCVA* best-corrected visual acuity, *CFT* central foveal thickness, *CST* central subfield thickness, *ETDRS* Early Treatment Diabetic Retinopathy Study, *IL* interleukin, *SD* standard deviation.

### IL-6 and VA outcomes in HARBOR

**Fig. 1:**
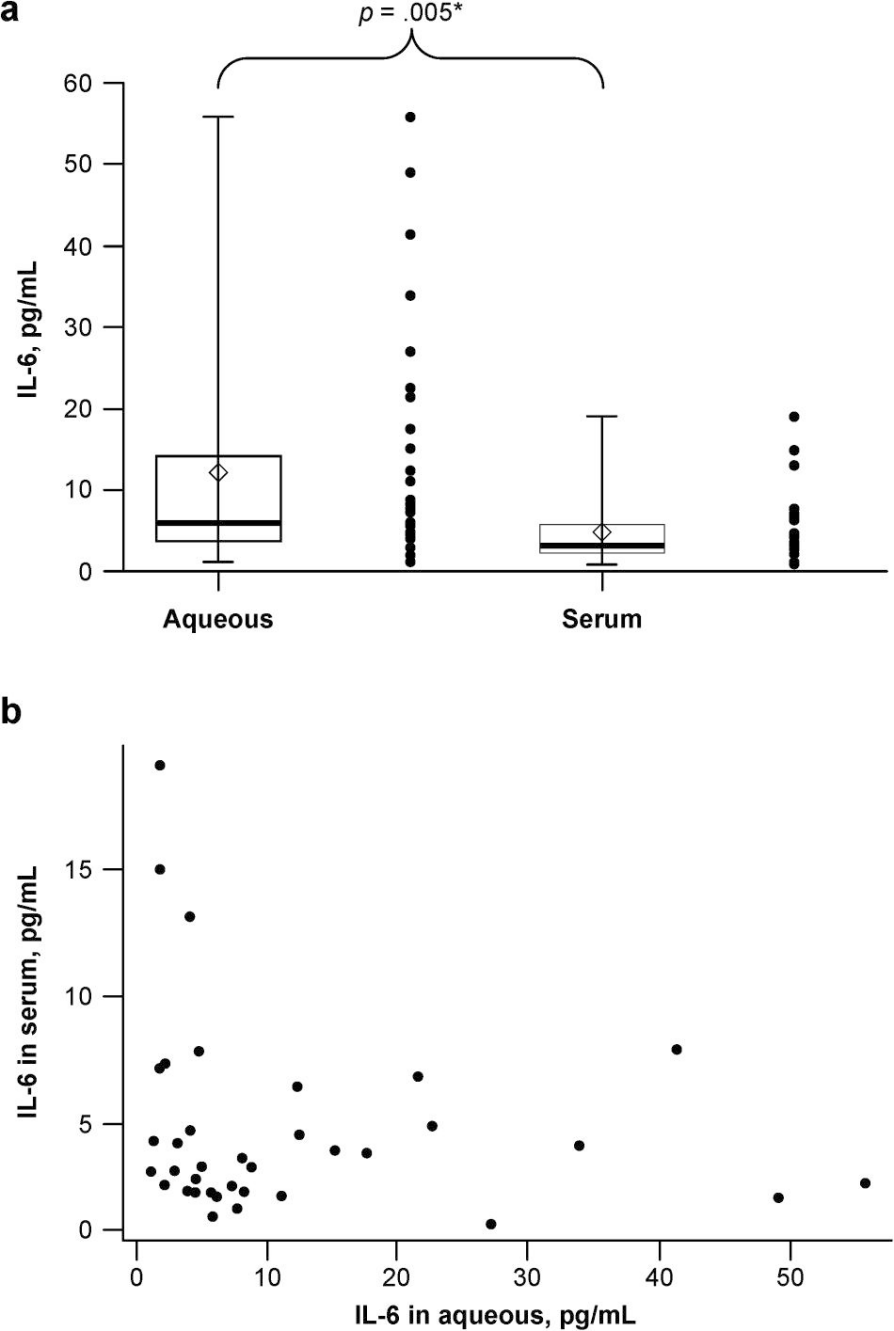
Interleukin (IL)-6 concentrations measured in aqueous humour and serum samples collected at month 2 of HARBOR. Box and whisker and scatter plots (**a**) and individual patient-level (**b**) interleukin (IL)-6 concentrations. In the box and whisker plot, the top and bottom lines of the box indicate the first and third quartiles, the centre line in the box indicates the median, the ends of the lines indicate the maxima and minima and the diamond indicates the mean. Pearson correlation coefficient: −0.1696. *Derived from Wilcoxon rank sum test comparing median aqueous humour and serum IL 6 concentrations.

At month 2, the median (quartile 1 [Q1]–Q3) IL-6 concentration in AH was 6.0 (3.7–13.9) pg/mL, which was greater than that of serum samples collected at the same time point (3.5 [2.3–5.7] pg/mL) (Fig. 1a). Moreover, patient-level data (Fig. 1b) showed a negative correlation between AH and serum IL-6 concentrations measured at month 2 (Pearson correlation coefficient, −0.1696).

**Fig. 2:**
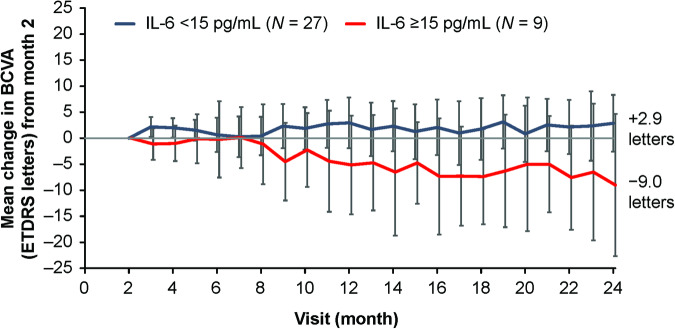
Change in best-corrected visual acuity (BCVA) over time in patients with high (≥15 pg/mL) and low (<15 pg/mL) aqueous humour interleukin (IL)-6 concentrations at month 2 of HARBOR. Data are mean (95% confidence interval). ETDRS Early Treatment Diabetic Retinopathy Study.

When patients were grouped by AH IL-6 concentration at month 2, we found that BCVA change through study end was improved in patients with low (bottom 75% of patients, <15 pg/mL) vs high (top 25% of patients, ≥15 pg/mL) IL-6 concentrations (Fig. 2). In the 27 out of 36 (75%) patients with an AH IL-6 concentration <15 pg/mL at month 2, the mean (95% confidence interval) BCVA change from baseline at month 2 was +3.4: (−0.4, 7.2) ETDRS letters, whereas the mean change from month 2 at month 24 was +2.9 (−2.6, 8.3) ETDRS letters. Conversely, in the 25% of patients who had an AH IL-6 concentration ≥15 pg/mL at month 2, the mean BCVA change from baseline at month 2 was +3.0 (−3.5, 9.5) ETDRS letters, whereas the mean change from month 2 at month 24 was −9.0 (−22.7, 4.7) ETDRS letters.

### IL-6 and VA outcomes in READ-3

**Fig. 3:**
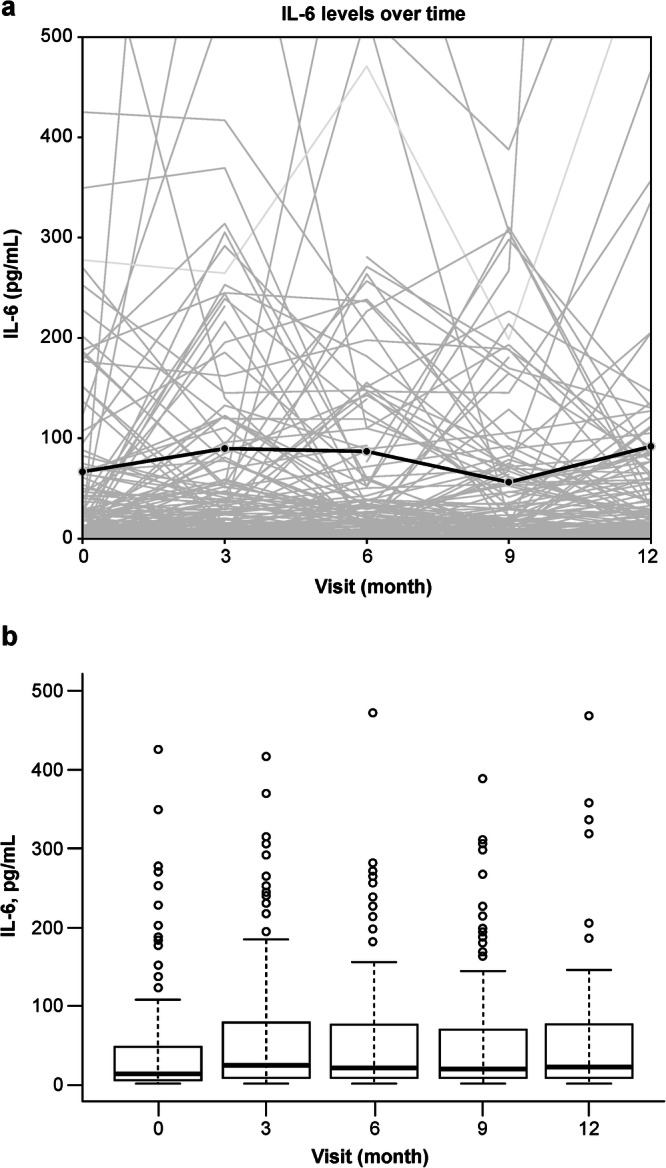
Aqueous humour interleukin (IL)-6 concentrations measured from baseline through month 12 of READ-3. Individual (grey lines in (**a**)), mean (black line in (**a**)), and box and whisker and scatter plots (**b**). In the box and whisker plot, the top and bottom lines of the box indicate the first and third quartiles, the centre line in the box indicates the median and the ends of the lines indicate the maxima and minima. The 0.5- and 2.0-mg ranibizumab doses were combined for analysis. A fixed range of 0–500 pg/mL for IL-6 concentration was used for improved visualisation in these figures.

AH IL-6 concentrations in patients with DMO measured at baseline through month 12 were highly variable, both within and between patients; however, IL-6 concentrations remained relatively stable over this period (Fig. 3, eFig. 1). The median (Q1–Q3) AH IL-6 concentration was 13.1 (4.6–46.5) pg/mL at baseline (*n* = 137). After three ranibizumab injections, the median AH IL-6 concentration increased to 25.0 (7.7–71.8) pg/mL (*n* = 131) and stabilised to 22.8 (6.6–76.7) pg/mL through month 12 (*n* = 123) (Fig. 3b).

**Fig. 4:**
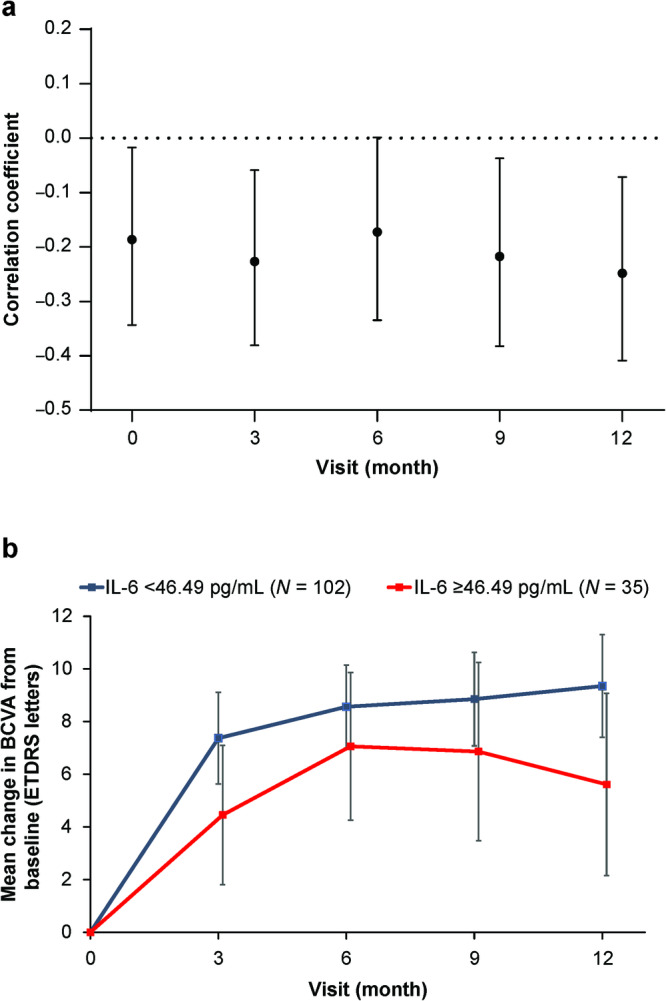
Correlation between interleukin (IL)-6 concentration at baseline and best-corrected visual acuity (BCVA) and change in BCVA over time stratified by baseline IL-6 concentration in READ-3. Correlation between aqueous humour IL-6 concentration at baseline (visit 0) and BCVA measured at each time point (**a**). Change in BCVA over time in patients with high (≥46.49 pg/mL) and low (<46.49 pg/mL) aqueous humour IL-6 concentrations at baseline (**b**). Data are **a** Spearman correlation coefficient (95% confidence interval [CI]) and **b** mean (95% confidence interval). ETDRS Early Treatment Diabetic Retinopathy Study.

Similar to the findings from HARBOR, higher AH IL-6 concentrations at baseline were associated with worse BCVA outcomes at month 12 in READ-3 (Fig. 4). Spearman correlation coefficients showed that BCVA measured at baseline and at months 3, 6, 9, and 12 were negatively correlated with AH IL-6 at baseline. Using the same method as in the HARBOR analyses, we observed a trend for lower BCVA gains from baseline through month 12 in patients with high (top 25% of patients, ≥46.49 pg/mL, *n* = 35) vs low (bottom 75% of patients, <46.49 pg/mL; *n* = 102) baseline AH IL-6 in READ-3. Mean BCVA (95% confidence interval) changes from baseline at month 12 were 5.6 (2.2–9.1) and 9.3 (7.4–11.3) ETDRS letters for high (≥46.49 pg/mL) vs low (<46.49 pg/mL) AH IL-6 concentrations at baseline, respectively.

## Discussion

There is mounting evidence that chronic inflammation plays a key role in the pathogenesis of retinal diseases, including nAMD and DMO [26–28]. There are numerous reports of increased concentrations of the pro-inflammatory cytokine IL-6 in the AH and vitreous humour of patients with nAMD and DMO [7–10, 16, 17, 23]. With this in mind, this study examined the relationship between intraocular IL-6 levels and VA outcomes achieved with ranibizumab treatment in the HARBOR and READ-3 trials.

In the HARBOR trial, there was no correlation between AH and serum IL-6 concentrations, with IL-6 levels being higher in the AH than in serum, suggesting local intraocular secretion. In addition, this finding also suggests that serum IL-6 concentrations may not be used as a surrogate for AH or vitreous IL-6 concentrations. The median AH IL-6 concentration measured in this study (6.0 pg/mL) is lower than the 10.1 pg/mL reported in patients with branch retinal vein occlusion [29] and is similar to the mean values reported in patients with treated nAMD (4.9 pg/mL) [30] and in patients with nAMD prior to treatment with anti-VEGF agents (5.9–6.5 pg/mL) [31, 32]. Other nAMD studies have reported average AH and serum IL-6 concentrations that are considerably higher than those measured in our analyses [10, 23, 30, 33]. This may be due to differences in IL-6 quantitation methods, patient characteristics, medications received, disease severity, and chronicity status. Nevertheless, our finding that intraocular IL-6 concentrations were significantly higher than serum concentrations is consistent with other observations reported in diabetic retinopathy and DMO [8, 34] and supports the hypothesis that intraocular IL-6 is locally derived in these conditions. The median serum IL-6 concentration of 3.5 pg/mL in HARBOR is similar to the median of 5.3 pg/mL reported for normal controls (age range, 24–68 years) and is lower than concentrations reported for patients with rheumatoid arthritis (34.1 pg/mL; age range, 20–78 years) [35], dermatomyositis (21.5 pg/mL; median age, 49 years), systemic lupus erythematosus (43.3 pg/mL; median age, 41 years) and Sjogren’s syndrome (26.7 pg/mL; median age, 44 years) [36].

Although we observed a high level of intra- and interpatient variability in AH IL-6 concentrations amongst READ-3 participants, averaged IL-6 concentrations remained relatively stable from baseline through month 12. This finding suggests that intraocular IL-6 concentrations were unaffected by anti-VEGF therapy in patients with DMO, a notable finding that has previously been reported in other studies (e.g., in patients with nAMD) [3]. Our hypothesis is also in keeping with previous research that identified IL-6 as an upstream mediator of VEGF in a murine model of choroidal neovascularisation [16]. The pathogenesis of DMO may be multifactorial, with the IL-6 and VEGF pathways acting independently of one another.

In both clinical trials (HARBOR and READ-3), we observed a trend for poorer VA outcomes amongst patients with higher AH IL-6 concentrations determined within the first 3 months of commencing ranibizumab treatment. These findings suggest that there may be a subgroup of patients who have high IL-6 concentrations and that those high IL-6 AH levels may act as a prognostic marker of response to therapy with anti-VEGF agents. Indeed, previous studies have also reported a correlation between AH IL-6 with anatomical responses to anti-VEGF therapy in patients with nAMD [3, 23]. Given the possible implications of elevated IL-6 in various ocular conditions, it may be plausible to consider IL-6 a therapeutic target, especially in patients with high IL-6 concentrations [37].

Several studies in other disease settings, including STOP-Uveitis (NCT01717170) and SATURN (NCT01900431), have demonstrated the therapeutic potential of systemic IL-6 receptor inhibitors for ocular conditions in which inflammation and IL-6 are predominantly implicated [14, 38]. The randomised, open-label STOP-Uveitis trial evaluated systemic tocilizumab in patients with non-infectious uveitis [14]. Results from the efficacy analysis showed a mean reduction in central macular thickness of −83.9 (136.1) µm at month 6 (*p* < 0.01) amongst all patients in the study. In SATURN, eyes with uveitic macular oedema demonstrated a reduction in central subfield thickness in response to systemic IL-6–R inhibition with subcutaneous sarilumab [38]. Specifically, amongst the subgroup of eyes with uveitic macular oedema (baseline central subfield thickness ≥300 µm), those in the sarilumab group had a mean reduction from baseline of 112.5 µm vs 1.8 µm in the placebo group. Note: the trial was not sufficiently powered for statistical analysis of this subgroup of patients.

Numerous studies suggest that DMO transition from a permeability-based disease, where a subset of patients generally respond to anti-VEGF monotherapy, to an inflammation-based multifactorial disease, where inflammatory mediators, including IL-6, may play an important role in mediating the underlying macular oedema [39, 40]. Indeed, the current body of evidence suggests that there is a proportion of patients who do not respond well to treatment with anti-VEGFs, presenting a so-called suboptimal or partial response, suggesting that VEGF may not be the sole driver or principal pathogenic mechanism in such patients. This provides an opportunity to individualise treatment (i.e., finding which patients can benefit the most from anti-VEGF therapy and which patients may need another specific targeted molecular approach to achieve the best structural and functional visual outcomes).

Both limitations and strengths should be considered when interpreting the results from these analyses. In HARBOR, because AH sampling was optional, only 36 of 1097 patients met the requirement to have matching serum and AH samples in order to be included in the analysis. Further, our study was limited to an analysis of serum and AH IL-6 concentrations measured at month 2, as baseline IL-6 measurements were not available in HARBOR, and patients had already received three injections before an AH sample was taken. Nevertheless, obtaining AH samples from a group of patients selected to represent a particular disease is challenging, and a sample size of 36 compares well with numerous published studies evaluating biomarkers in specific disease states [41, 42]. Unlike HARBOR, most of the patients enrolled in READ-3 (137/152) were included in the analyses reported herein.

Another potential limitation is that the assays used to analyse AH IL-6 used in both studies were different. However, we think the degree of correlation between the two assays is high. Further, there were differences between trials in the inclusion of treatment-naïve and previously treated patients (HARBOR only included treatment-naïve patients, whereas READ-3 included previously treated and treatment-naïve patients). Nevertheless, despite the different assays and populations included, the findings are consistent: high levels of IL-6 in AH may indicate less response to anti-VEGF therapy. Further studies are warranted to prove this hypothesis in various retinal diseases and build a future of personalised treatment in ophthalmology.

In conclusion, this post hoc analysis of HARBOR and READ-3 found that higher concentrations of AH IL-6 may predict suboptimal visual responses to anti-VEGF therapy in patients with nAMD and DMO. Our data highlight the key role of chronic inflammation in the pathogeneses of nAMD and DMO. Intraocular levels of IL-6 may help predict response to anti-VEGF standard of care in the setting of retinal diseases. IL-6-targeted inhibition may be a more suitable strategy for those patients with a predominantly inflammatory disease profile.

## Summary

### What was known before

- Previous studies have demonstrated correlations between aqueous humour interleukin-6 (IL-6) concentrations and changes in central retinal thickness in response to anti-vascular endothelial growth factor therapy (VEGF).

### What this study adds

- Results from our post hoc analyses of data from the HARBOR and READ-3 trials indicate that intraocular IL-6 levels may predict anti-VEGF therapy response in patients with neovascular age-related macular degeneration and diabetic macular oedema.

### Supplementary information

Individual aqueous humour interleukin (IL)-6 concentrations measured from baseline through month 12 of READ-3 (with full range of data)

## Data Availability

For HARBOR, qualified researchers may request access to individual patient-level clinical data through a data request platform. At the time of writing, this request platform is Vivli (https://vivli.org/ourmember/roche/). For up-to-date details on Roche’s Global Policy on the Sharing of Clinical Information and how to request access to related clinical study documents, see here: https://go.roche.com/data_sharing. Anonymised records for individual patients across more than 1 data source external to Roche cannot, and should not, be linked due to a potential increase in risk of patient re-identification.
