# Supplementary material for: Aqueous humour interleukin-6 and vision outcomes with anti-vascular endothelial growth factor therapy
Source: Eye (Lond). 2024 Apr 15;38(9):1755–61. doi: 10.1038/s41433-024-03015-2 (PMC11156666; doi:10.1038/s41433-024-03015-2)

**Supplementary Information**

**eFig. 1** Individual aqueous humour interleukin (IL)-6 concentrations measured from baseline through month 12 of READ-3 (with full range of data).

**eFig. 1** Individual aqueous humour interleukin (IL)-6 concentrations measured from baseline through month 12 of READ-3 (with full range of data). The 0.5- and 2.0-mg ranibizumab doses were combined for analysis.


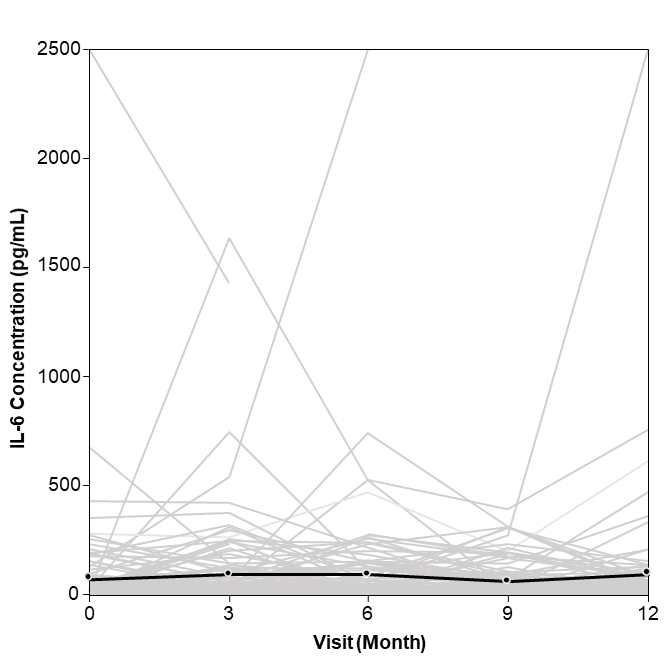

Supplement: Supplementary file 1 — Individual aqueous humour interleukin (IL)-6 concentrations measured from baseline through month 12 of READ-3 (with full range of data) [file 41433_2024_3015_MOESM1_ESM.docx]
